# Abdominal organs' microcirculation dysfunction sequence in severe sepsis by SDF microscopy and histology

**DOI:** 10.1186/cc11780

**Published:** 2012-11-14

**Authors:** J Almeida-Filho, AMA Liberatore, RC Tedesco, EC Del-Massa, IHJ Koh

**Affiliations:** 1 Federal University Foundation of Vale do São Francisco, São Paulo, Brazil; 2 Federal University of São Paulo, Brazil

## Background

The microcirculation dysfunction sequence in sepsis has not been well acknowledged to support therapy and prognosis. Herein, the abdominal organs' microcirculation and their perivascular tissue derangements captured by SDF microscopy were correlated with the whole organ histological findings in severe sepsis.

## Methods

Adult Wistar-EPM rats (*n *= 54) were distributed into: sepsis group (*n *= 36), animals submitted to 10^9 ^CFU/ml *Escherichia coli *inoculation through the jugular vein; and sham group (*n *= 18), animals with physiologic saline 0.9%. At 0, 2, 6 and 24 hours, liver, kidney and ileum microvascular and perivascular images were monitored by SDF *in vivo *and also by histology.

## Results

**Figure 1:**
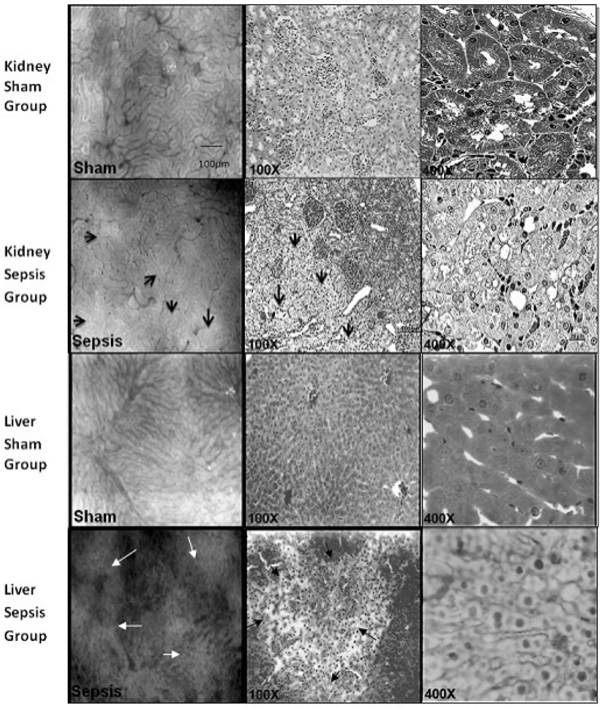
**Organ SDF and histological findings in sham and sepsis groups at the 6-hour period**. Arrows indicate vascular and perivascular tissue derangements.

The liver and kidney surface SDF images showed that microcirculation and perivascular tissue alterations are a concomitant event, which were a focal event initially that turned progressively generalized in proportion to sepsis worsening. Vascular patterns were from complete absence to hyperflow and narrowed to dilated venules, showing that altered and nonaltered microvessels occur simultaneously in sepsis. The expansion of perivascular tissue fuzziness with narrowed or vanished microvessels in sepsis suggested local dysfunction in progression. Confronting these areas with histology, the enlarged perivascular areas were composed mostly of cytoplasm edema at the early sepsis phase and of varying stages of the cell necrosis process at the further periods. These processes were very similar in both the liver and kidney and occurred throughout the organ, showing that the surface findings can be extensive to deeper areas (Figure 1). In contrast, the small bowel SDF images showed that gut surface microcirculation was composed primarily of high-flow capillaries and only the vascular density was focally reduced in sepsis; besides, the perivascular tissues could not be identified, showing that the vascular and tissue architectural pattern in the gut differs substantially from solid organs, suggesting a different pathophysiology response of the intestinal microcirculation in sepsis. The gut histology showed generalized cell edema and varying necrosis phases in muscle layers of the intestine in sepsis, and such events could not be suspected by SDF.

## Conclusion

Organ dysfunction in sepsis is better detectable in solid organs by SDF imaging as compared with gut muscular compartment. These results demonstrated the importance of the solid organ SDF monitoring in experimental sepsis treatment studies.

